# Draft Genome Sequences of Six Enterohepatic *Helicobacter* Species Isolated from Humans and One from Rhesus Macaques

**DOI:** 10.1128/genomeA.00857-14

**Published:** 2014-09-11

**Authors:** Zeli Shen, Alexander Sheh, Sarah K. Young, Amr Abouelliel, Doyle V. Ward, Ashlee M. Earl, James G. Fox

**Affiliations:** a Division of Comparative Medicine, Massachusetts Institute of Technology, Cambridge, Massachusetts, USA; b Broad Institute of MIT and Harvard, Cambridge, Massachusetts, USA; c Department of Biological Engineering, Massachusetts Institute of Technology, Cambridge, Massachusetts, USA

## Abstract

Draft genome sequences of seven enterohepatic *Helicobacter* species, *H. bilis, H. canadensis, H. canis, H. cinaedi, H. winghamensis, H. pullorum*, and *H. macacae*, are presented. These isolates were obtained from clinical patients and a nonhuman primate. Due to potential zoonotic risks, we characterized antibiotic resistance markers and *Helicobacter* virulence factors.

## GENOME ANNOUNCEMENT

Enterohepatic *Helicobacter* species (EHS) are Gram-negative, microaerophilic, spiral-shaped bacteria that colonize the mucosa of the gastrointestinal tract and/or liver of mammals, including humans, and birds (1, 2). Seven EHS species (*H. canadensis* [3], *H. canis* [4], *H. cinaedi* [5], *H. fennelliae* [5], *H. winghamensis* [6], *H. pullorum* [7], *and H. bilis* [8–10]) have been isolated from both healthy humans and patients with wide-ranging symptoms, such as diarrhea, fever, proctocolitis, enteritis, meningitis, bacteremia, septic arthritis, cholecystitis, elevated liver enzymes, and hepatomegaly (2, 3, 5, 7, 11–26). Unlike *H. pylori*, EHS can infect animal reservoirs, such as dogs, cats, geese, wild and laboratory rodents, rhesus macaques, hamsters, gerbils, guinea fowl, and chickens, highlighting the risk of zoonotic infections (7, 26–43).

In this report, we announce the whole-genome sequencing of seven EHS, including *H. bilis* ATCC 43879, *H. canis* NCTC 12740, *H. canadensis* NCTC 13241, *H. cinaedi* CCUG 18818, *H. macacae* CCUG 55313^T^, *H. pullorum* MIT 98-5489, and *H. winghamensis* ATCC BAA-430. While *H. macacae* was isolated from both healthy and diseased rhesus macaques (42, 43), the six remaining strains were human isolates. Isolates were sequenced using 454 or Illumina sequencing technology. 454 sequencing reads were assembled using Newbler assembler v. 2.1 and annotated as previously described (44). Illumina reads were assembled using ALLPATHS version R39721 (45) and annotated as performed previously (46).

Due to the potential of zoonotic disease, antibiotic resistance and the presence of virulence factors were evaluated. *H. canadensis* and *H. pullorum* isolates are clarithromycin resistant, which may be mediated by a mutation in the 23S *rRNA* gene (A2143G) in *H. pullorum*, but a mutation conferring clarithromycin resistance to *H. canadensis* remains undetermined. *H. canadensis, H. pullorum, H. bilis*, and *H. winghamensis* isolates are rifampin resistant, and four missense mutations in RpoB (M517L, V557A, A561V, and T593A) were shared by *H. canadensis, H. pullorum*, and *H. winghamensis*. However, these mutations were not found in the *H. bilis rpoB*. *H. canadensis, H. pullorum, H. bilis*, and *H. cinaedi* are tetracycline resistant, and triple-base-pair 16S *rRNA* gene mutations in AGA926-928, which confer tetracycline resistance (47), were found in *H. pullorum* (TGA), *H. bilis* (AGC), and *H. cinaedi* (GTA), but not in *H. canadensis*. *H. bilis*, *H. canadensis*, and *H. pullorum* are ciprofloxacin resistant, and individual missense mutations (V89I, A87I, and A87I, respectively) were detected in gyrA.

The seven EHS isolates contained ferroxidases with homology to the *H. pylori napA* gene that encodes the neutrophil-activating protein (48), as well as the HtrA protease, which cleaves E-cadherin (49). *H. canis, H. cinaedi*, and *H. pullorum* possess cytolethal distending toxin (cdt) genes A, B, and C, while *H. bilis* and *H. winghamensis* possess *cdtB* and *cdtC*. Gene clusters with the *icmF*, *hcp*, and *vrgG* genes, encoding type VI secretion systems, were found in *H. bilis, H. cinaedi*, and *H. pullorum*, and may play a role in disease (50). The gamma-glutamyl transpeptidase (ggt) gene, an *H. pylori* virulence factor (51), was also found in *H. bilis* and *H. canis*. More detailed characterizations of these EHS isolates are forthcoming.

### Nucleotide sequence accession numbers.

Genome sequences have been submitted to GenBank under the accession numbers listed in Table 1.

**TABLE 1:** Genome characteristics and accession numbers of seven enterohepatic *Helicobacter* species^*a*^

Strain	GenBank accession no.	Other available sequenced genomes (strain [accession no.] [reference])^*c*^	Genome size (Mb)	Contig count	Fold coverage	GC (%)	Gene count
*H. bilis* ATCC 43879 (4)^*b*^	ACDN00000000.2	WiWa (AQFW00000000.1)&, ATCC 51630 (JMKW00000000.1)&	2.53	77	18×	34.79	2,338
*H. canis* NCTC 12740 (I)^*b*^	AZJJ00000000.1		1.93	7	143×	45.00	1,800
*H. canadensis* NCTC 13241 (MIT 98-5491) (4)^*b*^	ABQS00000000.1	MIT 98-5491 (ACSF00000000.1) (52)	1.63	126	49×	33.66	1,628
*H. cinaedi* CCUG 18818 (4)^*b*^	ABQT00000000.1	PAGU611 (AP012344.1) (53), ATCC BAA-847 (AP012492.1) (54)	2.21	96	39×	38.46	2,374
*H. macacae* CCUG 55313^T^ (MIT 99-5501) (I)^*b*^	AZJI00000000.1		2.37	12	141×	40.60	1,946
*H. pullorum* MIT 98-5489 (4)^*b*^	ABQU00000000.1		1.95	131	60×	34.15	2,008
*H. winghamensis* ATCC BAA-430 (4)^*b*^	ACDO00000000.1		1.69	55	61×	35.49	1,639

a *H. fennelliae* MRY12-0050, a human isolate, has also been sequenced (55).

b Sequenced as part of the Human Microbiome U54 initiative, Broad Institute (http://broadinstitute.org); (I), sequenced using Illumina; (4), sequenced using 454.

c &, unpublished genome.
